# Screening and Identification of Key Genes for Activation of Islet Stellate Cell

**DOI:** 10.3389/fendo.2021.695467

**Published:** 2021-09-09

**Authors:** Xiaohang Wang, Vladmir Carvalho, Qianqian Wang, Jinbang Wang, Tingting Li, Yang Chen, Chengming Ni, Lili Liu, Yang Yuan, Shanhu Qiu, Zilin Sun

**Affiliations:** ^1^Department of Endocrinology, Zhongda Hospital, Institute of Diabetes, School of Medicine, Southeast University, Nanjing, China; ^2^Department of General Practice, Zhongda Hospital, Institute of Diabetes, School of Medicine, Southeast University, Nanjing, China

**Keywords:** islet stellate cell, RNA-seq, Fos, Pdpn, Bad, islet fibrosis

## Abstract

**Background:**

It has been demonstrated that activated islet stellate cells (ISCs) play a critical role in islet fibrogenesis and significantly contribute to the progression of type 2 diabetes mellitus. However, the key molecules responsible for ISCs activation have not yet been determined. This study aimed to identify the potential key genes involved in diabetes-induced activation of ISCs.

**Method:**

Stellate cells were isolated from three 10-week-old healthy male Wistar rats and three Goto-Kakizaki (GK) rats. Cells from each rat were primary cultured under the same condition. A Genome-wide transcriptional sequence of stellate cells was generated using the Hiseq3000 platform. The identified differentially expressed genes were validated using quantitative real-time PCR and western blotting in GK rats, high fat diet (HFD) rats, and their controls.

**Results:**

A total of 204 differentially expressed genes (DEGs) between GK. ISCs and Wistar ISCs (W.ISCs) were identified, accounting for 0.58% of all the 35,362 genes detected. After the Gene Ontology (GO) and Kyoto encyclopedia of genes and genomes (KEGG) enrichment analyses, the mRNA levels of these genes were further confirmed by real-time PCR in cultured ISCs. We then selected *Fos*, *Pdpn*, *Bad* as the potential key genes for diabetes-induced activation of ISCs. Finally, we confirmed the protein expression levels of FOS, podoplanin, and Bad by western blotting and immunofluorescence in GK rats, HFD rats, and their controls. The results showed that the expression level of FOS was significantly decreased, while podoplanin and Bad were significantly increased in GK.ISCs and HFD rats compared with controls, which were consistent with the expression of α-smooth muscle actin.

**Conclusions:**

A total of 204 DEGs were found between the GK.ISCs and W.ISCs. After validating the expression of potential key genes from GK rats and HFD rats, *Fos*, *Pdpn*, and *Bad* might be potential key genes involved in diabetes-induced activation of ISCs.

## Introduction

Type 2 diabetes mellitus (T2DM) has become a public health burden and is one of the leading causes of morbidity and mortality worldwide (1). T2DM is characterized by inadequate beta-cell response due to the progressive insulin resistance that typically accompanies with physical inactivity and weight gain (2). Due to the rapid increase in the morbidity of T2DM, it is of importance to have a better understanding of the pathophysiology of T2DM. Islet fibrosis in T2DM has attracted increasing research attention and has been intensively studied (3–6). Islet fibrosis leads to a decreased β cell mass and compromises insulin secretion from the pancreas, while alleviating islet fibrosis may restore β-cell function and delay the progression of diabetes.

In the exocrine pancreas, it has been verified that pancreatic stellate cells (PSCs) play a critical role in the pathogenesis of pancreatic fibrosis in chronic pancreatitis and pancreatic cancer (7). The pathogenesis of islet fibrosis has not yet been fully clarified, although the profibrogenic effects of PSCs in islet fibrogenesis have been demonstrated in both rodent animal models and human patients with T2DM (8–13). Recently our group identified a distinct population of stellate cells, which could be expanded from isolated islets in both humans and animals (14–16). They were named as islet stellate cells (ISCs), showing some similarities as classical PSCs. For example, they all express α-smooth muscle actin (α-SMA) - a recognized biomarker of activation, nestin, glial fibrillary acidic protein (GFAP), etc. However, they exhibit differences in cell activation, proliferation, migration, and collagen synthesis. Through transcriptome sequencing, we also found that ISCs and PSCs have similar characteristics (17). Stellate cells stay quiescent in general. However, when suffering stimulation, such as chronic inflammation and oxidative stress, they are activated, turning into myo-fibroblast cells. Activated stellate cells lose their vitamin A-storing lipid droplets in their cytoplasm, expressing the cytoskeletal marker protein α-SMA and extracellular matrix (ECM) components such as collagens and fibronectin. They also produce cytokines and chemokines such as transforming growth factor β (TGF-β) and platelet-derived growth factor (PDGF) and increase proliferation and migration activity.

It was found that the level of infiltration and activation of stellate cells in the pancreatic islets were increased in the process of islet fibrosis in Otsuka Long-Evans Tokushima Fatty (OLETF) rats and Goto-Kakizaki (GK) rats, one of the best characterized animal models of spontaneous T2DM (8, 18). In patients with T2DM and cystic fibrosis-related diabetes, a mass of activated stellate cells in pancreatic islets was also observed (19, 20). In agreement with their work, our group found that ISC activation was significantly increased in obese rats induced by high-fat diet. However, inhibition of stellate cell activity reduces cellular inflammation and restores the function of islets (8, 13, 18). The use of Pancreatic Stone Protein (PSP) in diabetic mice can inhibit the exogenous rate, activation, cell proliferation, migration, extracellular matrix synthesis of ISCs, and improve ISC-induced islet cell dysfunction in diabetic conditions (21). PSP can relieve the toxic effects of ISCs on β cell and reduce β cell apoptosis. Thus, it has been verified that ISCs are already activated in T2DM and play an important role in islet fibrosis, promoting T2DM progression. However, the mechanism underlying the activation of ISCs remains unclear. Therefore, sorting out the key molecules that enhance the biological activity of ISCs in diabetes is essential in implementing interventions in reducing islet fibrosis and delaying the progression of diabetes.

In this study, we isolated ISCs from islets of GK rats and Wistar rats, and performed RNA-seq. Then, we analyzed the differentially expressed genes through Gene Ontology (GO) and Kyoto encyclopedia of genes and genomes (KEGG) enrichment analyses to identify genes that may cause ISC activation. Finally, we validated the expression levels of differentially expressed genes by real-time PCR, western boltting, and immunofluorescence in histologic sections to identify the potential key genes involved in diabetes-induced activation of ISCs.

## Materials and Methods

### Animals

Three 10-week-old healthy male Wistar rats and three GK rats were chosen for this study, and given access to food and water ad libitum. After a one-week acclimation period, the rats were given anesthesia and sacrificed to collect pancreas. The Wistar rats were numbered as A, B and C, and the GK rats were as D, E and F. ISCs from Wistar rats were named as A12, B12 and C12, respectively. Similarly, the ISCs from GK rats were named as D12, E12 and F12, respectively. Sprague-Dawley (SD) rats were used for additional validation of the sequencing. Rats were divided into a normal diet (ND) group and a high-fat diet (HFD) group as described previously (22). Housing and animal experiments were approved by Southeast University Animal Care and Use Committee according to institutional guidelines and national animal welfare.

### Isolation and Culture of ISCs

Procedures were conducted as previously described (23). Briefly, pancreas tissues from rats were digested with collagenase P (1 mg/mL, w/v) (Sigma, St. Louis, MO, USA). Then the islets were purified by handpicking under a stereomicroscope. Freshly isolated islets were subsequently cultured in culture dishes with Dulbecco’s modified Eagle’s medium/Ham’s F12 (DMEM/F12, Gibco) with 10% fetal bovine serum at 37°C (95% air/5% CO_2_). ISCs began to grow out of islets after 2 days. When the cultures were nearly confluent with stellate cells, cells were harvested with trypsin and re-cultured after diluting the cell suspension twice. These experiments were performed using ISCs at three passages after isolation. The purity of the isolated ISCs was determined by staining for desmin. Only isolations with purity >95% were used for further experiments.

### mRNA Library Construction and Sequencing

The details for mRNA library construction and sequencing are shown in **Supplementary File 1**. The sequencing data generated in this study were deposited in the National Center for Biotechnology Information (NCBI) Sequence Read Archive database, and the bioproject accession number is PRJNA731990 (http://www.ncbi.nlm.nih.gov/sra/).

### Functional Enrichment Analysis

The detailed approaches for functional enrichment analysis are provided in **Supplementary File 1**.

### Quantitative Real-Time PCR

Total RNA from two groups were isolated using a rapid extraction method (TRI-Reagent, Invitrogen). For the cDNA synthesis, 1 μg of total RNA of each sample was reverse transcribed using HiScript II Q RT SuperMix for qPCR (R223-01; Vazyme, China). Real-time PCR was performed on cDNA samples using the FastStart Universal SYBR Green Master (Roche) on Step One Plus system (Applied Biosystems, Foster City, CA, USA). Primers are described in **Table S1**. The PCR settings used included denaturation (95°C for 2 min) and amplification steps repeated 40 times (95°C for 15s, 55°C for 30s, 72°C for 30s, and acquisition temperature for 15s). Analysis was conducted using the sequence detection software supplied with the instrument. For each sample, the delta delta Cycle of Threshold (ddCT) (crossing point) values were calculated as the Ct of the target gene minus the Ct of the β-actin or 18s rRNA, assuming PCR efficiency equals to 1. Gene expression was derived according to the equation 2^–ddCt^; changes in gene expression are expressed relative to levels of the other group.

### Western Blotting

PVDF membranes containing electrophoretically separated proteins from cells were probed with rabbit antibodies against Podoplanin (EPR7073; Abcam), Fos (sc-166940; Santa Cruz), Bad (sc-8044; Santa Cruz), α-SMA (bs-0189R; Bioss), GAPDH (60004-1-lg; Proteintech), treated with peroxidase-conjugated goat anti-mouse or anti-rabbit IgG secondary antibody (BL001A; BL003A; Biosharp), and then visualized by enhanced Chemiluminescent horseradish peroxidase (HRP) Substrate (Millipore).

### Immunofluorescence

The sections of pancreatic tissues were blocked by 10% goat serum (Servicebio, Wuhan, China) and incubated with primary antibodies at 4°C overnight. The primary antibodies used in this experiment were: rabbit anti-insulin (1:200; bs-0056R; Bioss), mouse anti-insulin (1:200; BM0080; Boster), rabbit anti-podoplanin (1:200; A01124-2; Boster), mouse anti-Fos (1:100; sc-166940; Santa Cruz), mouse anti-Bad (1:100; sc-8044; Santa Cruz). The secondary antibodies (SA00013-1, SA00013-2, SA00013-3, SA00013-4) for immunostaining were all purchased from Proteintech. 4′,6′-diamidino-2-phenylindole (DAPI; Servicebio) was used for nuclei staining. Olympus FV1000 confocal microscopy system was used for images capture.

### Statistical Analysis

All data are presented as means ± standard error of the means (SEM). Student’s t-test was used to assess the differences between groups. Statistical significance was set at *P* < 0.05 (IBM SPSS Statistics 22). All statistical analyses were performed using GraphPad Prism (Version 7.0.4).

## Results

### Evaluation of Gene Expression Profiles

In order to obtain the coverage area and coverage depth of the sequencing data, we used Tophat software (Version 2.0.4) for analysis. The effective numbers of reads generated from each sample ranged from 50,777,686 to 64,829,046. Most of the readings in each sample can be specifically aligned with the reference Rattus genome sequence, and the percentage of aligned reads can range from 84.64% to 85.18% (**Table 1**). Then we performed the statistical analysis of the distribution of the mapped readings for each sample (**Figure S1**). As shown in **Figure S1**, the percentage of exonic distribution ranged from 94.21% to 96.12%. And the intronic distribution varied from 2.21% to 4.00%. The remaining area was intergenic distributed because of the incompletion of the genetic annotation, which may lead to the detection of new genes or new long non-coding RNA. The total number of detected genes from all the samples was 35362. Specifically, the number of detected genes from each sample was (A12:17984, B12:18401, C12:18304, D12:17951, E12:18579, F12:18451).

**Table 1:** The comparison result with clean data and Rattus genome sequences.

Mapped statistics	A12	B12	C12	D12	E12	F12
Total reads	50,777,686 (100%)	53,749,152 (100%)	54,536,444 (100%)	53,338,794 (100%)	64,829,046 (100%)	56,975,832 (100%)
Total mapped	46,188,430 (90.96%)	49,072,733 (91.3%)	49,552,463 (90.86%)	48,874,364 (91.63%)	59,411,895 (91.64%)	52,352,276 (91.89%)
Multiple mapped	3,172,290 (6.25%)	3,288,591 (6.12%)	3,290,626 (6.03%)	3,617,347 (6.78%)	4,195,396 (6.47%)	4,128,531 (7.25%)
Uniquely mapped	43,016,140 (84.71%)	45,784,142 (85.18%)	46,261,837 (84.83%)	45,257,017 (84.85%)	55,216,499 (85.17%)	48,223,745 (84.64%)

A12, B12 and C12 are W.ISCs from three Wistar rats. D12, E12, F12 are GK.ISCs from three GK rats.

### Analysis of Differentially Expressed Genes

**Figure 1:**
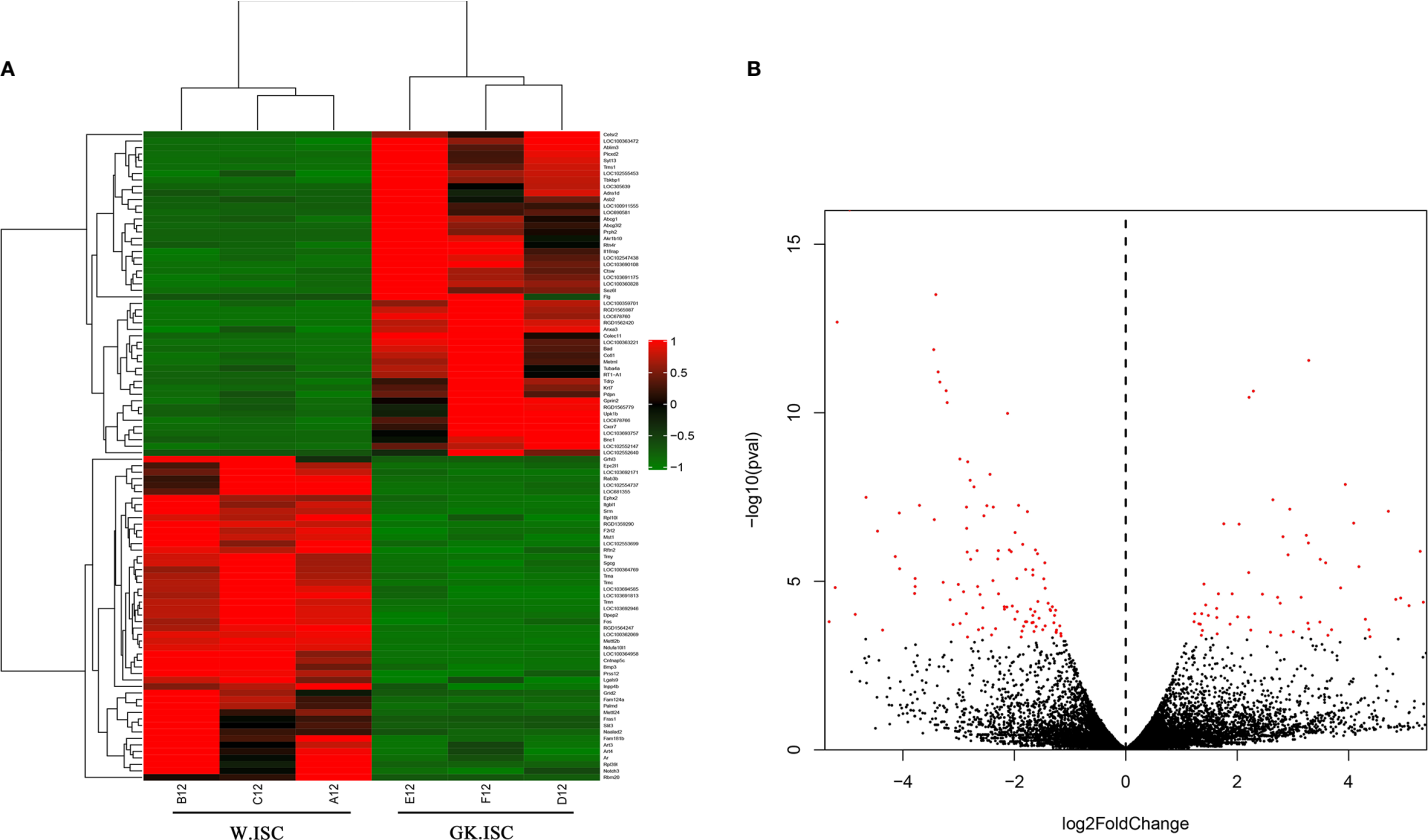
Heatmap and Volcano plot showing DEGs in W.ISCs and GK.ISCs. **(A)** Heatmap shows the hierarchically clustered top100 differentially expressed genes. Up-regulated levels of gene expression are displayed as red bars while down-regulated levels are displayed as green bars. **(B)** Volcano plot shows the overall distribution of DEGs. Genes with fold change > 2 and statistical significance are marked with red dots.

In order to clarify the differentially expressed genes (DEGs) between Wistar-ISCs and GK-ISCs samples, we performed a negative binomial distribution test (NB) to test the difference in the number of reads. The genes with an adjusted *P-*value <0.05 (Q<0.05) and a fold change of more than two were counted. There were 204 significant differentially expressed genes between W.ISCs and GK.ISCs. Compared with W.ISCs, 73 genes were significantly up-regulated, and 131 genes significantly down-regulated in the GK.ISCs. The gene expression profiles were presented in a cluster heatmap and a volcano figure (**Figure 1**). The list of the 204 DEGs is presented in **Table S2**.

### KEGG Biological Pathway Enrichment Analysis

**Figure 2:**
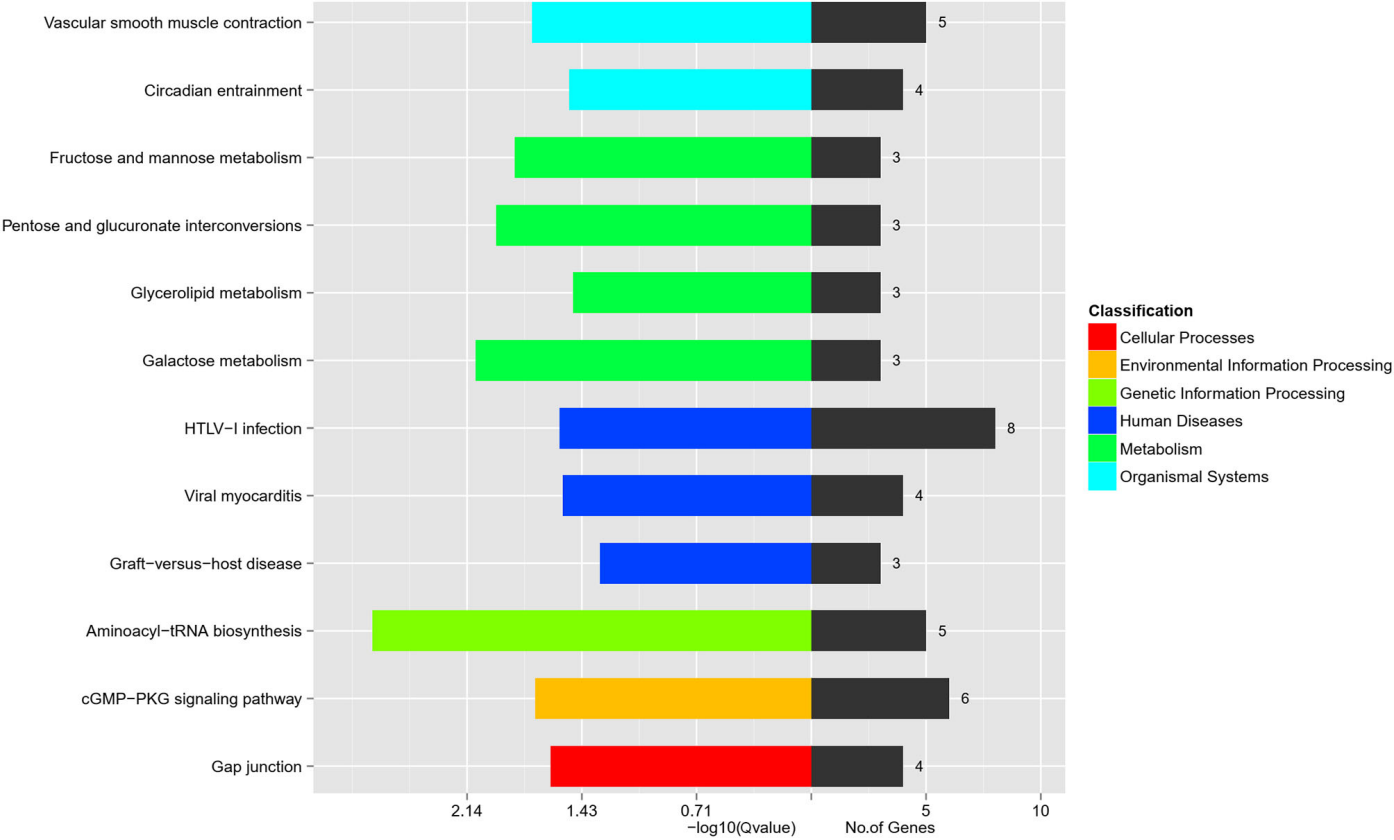
KEGG pathway analysis of DEGs in W.ISCs and GK.ISCs. The most enrichment pathways in different classifications.

The biological pathway analysis referred to the KEGG database (http://www.genome.jp/) The top 3 most enriched pathways were Aminoacyl-tRNA biosynthesis, Galactose metabolism, and Pentose and glucuronate interconversions (*P* < 0.05) (**Figure 2**). The genes involved in the top 3 KEGG pathways are presented in **Table 2**. The aminoacyl-tRNA biosynthesis suggests that there is a significant difference between GK.ISCs and W.ISCs in protein synthesis. This might be attributable to the differences in proliferation, migration rates, protein synthesis, and secretion between activated and quiescent ISCs. The Galactose metabolism and Pentose and glucuronate interconversions revealed that there is a difference in glucose metabolism between ISCs in diabetes and normal conditions.

**Table 2:** The top 3 KEGG pathways involved in W.ISCs and GK.ISCs.

Term	database	ID	*P* value	Gene
Aminoacyl-tRNA biosynthesis	KEGG PATHWAY	rno00970	0.001862	*Trns1*; *Trny*; *Trnc*; *Trnn*; *Trna*
Galactose metabolism	KEGG PATHWAY	rno00052	0.008129	*Akr1b8*; *Akr1b10*; *Akr1b1*
Pentose and glucuronate interconversions	KEGG PATHWAY	rno00040	0.010964	*Akr1b8*; *Akr1b10*; *Akr1b1*

### The Gene Ontology Functional Enrichment Analysis of DEGs

**Figure 3:**
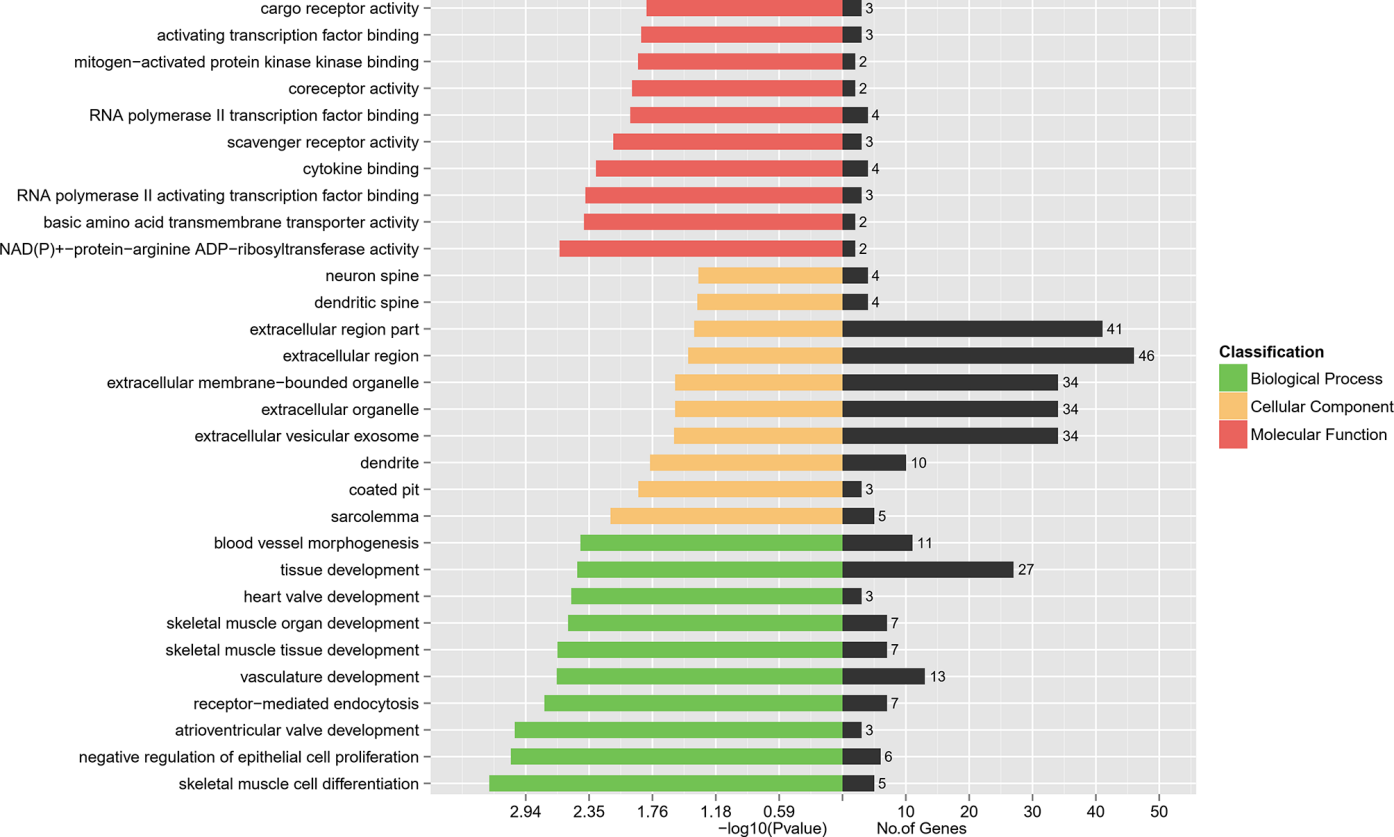
DEGs’ GO enrichment classification. The red bars represent the Molecular Function. The yellow bars represent the Cellular Component while the green represent the Biological Process.

To interpret the possible biological roles of the DEGs in W.ISCs and GK.ISCs, we made function annotation for each gene through GO functional enrichment analysis. The 204 common genes were enriched in 2966 GO terms. As for the biological process (BP), the genes were mainly enriched in skeletal muscle cell differentiation and negative regulation of epithelial cell proliferation. In regard to cellular component (CC), condensed sarcolemma and coated pit were mainly enriched. Regarding molecular function (MF), the genes were mainly enriched in NAD(P)^+^-protein-arginine ADP-ribosyltransferase activity and basic amino acid transmembrane transporter activity. **Figure 3** lists the main GO terms that were enriched in the differentially regulated genes. The difference between W.ISCs and GK.ISCs may involve changes in other molecular processes. Consequently, other GO terms were also annotated during the pathological changes. We only focused on the GO terms related to cell proliferation, cell migration, and ECM components synthesis as these processes are crucial for changes of ISCs in diabetes. These involved GO terms are presented in **Table 3**. *Ptprk*, *Figf*, *Timp1*, *Pitx2*, *Acvrl1*, and *Pdpn* played roles in both cell migration and cell proliferation. *Ptprk*, *Fos*, *Cav2*, and *Acvrl1* participated in transforming growth factor beta receptor signaling pathway, which is a classic pro-fibrosis pathway. Moreover, *Arrb1* and *Ksr1* were involved in mitogen-activated protein kinase signaling pathway, which may influence cell proliferation, differentiation, transformation, and apoptosis in activated ISCs.

**Table 3:** The main GO terms involved in activation of ISCs.

Term	*P* value	Gene Names
Biological Process (BP)		
positive regulation of cell proliferation	0.012282674	*Notch3*; *Akr1b1*; *Htr2b*; *Figf*; *Hey2*; *Crip2*; *Gas1*; *Timp1*; *Egr1*; *Pitx2*; *Acvrl1*; *Bad*; *Ar*; *Cav2*
regulation of cell migration	0.022041922	*Ptprk*; *Spata13*; *Mylk*; *Figf*; *Unc5c*; *Anxa3*; *Timp1*; *Pitx2*; *Acvrl1*; *Pdpn*
cell proliferation	0.022494784	*Figf*; *Rerg*; *Egr1*; *Pdpn*; *Bad*; *Slit3*; *Wfdc1*; *Ptprk*; *Notch3*; *Akr1b1*; *Htr2b*; *Hey2*; *Timp1*; *Gas1*; *Dock8*; *Pitx2*; *Acvrl1*; *Ar*; *Wisp2*; *Megf10*; *Crip2*; *Cav2*; *Jup*
transforming growth factor beta receptor signaling pathway	0.030074189	*Ptprk*; *Fos*; *Cav2*; *Acvrl1*
Cellular Component (CC)		
extracellular vesicular exosome	0.02741718	*Pcbp3*; *Upk1b*; *Fzd4*; *Galnt16*; *Islr*; *Cotl1*; *Mylk*; *LOC102555453*; *LOC681355*; *Trabd2b*; *Metrnl*; *Bmp3*; *Hebp2*; *Akr1e2*; *Tuba4a*; *Art3*; *Akr1b1*; *Timp1*; *Anxa3*; *Sepp1*; *Lgals9*; *Krt7*; *Flot1*; *Prps2*; *Rtn4r*; *Mst1*; *Ptgr1*; *Tnik*; *Gdf15*; *Wisp2*; *Ephx2*; *Rab3b*; *Col6a2*; *Jup*
Molecular Function (MF)		
cytokine binding	0.005159	*Acvrl1*; *Fzd4*; *Cxcr7*; *Grem2*
mitogen-activated protein kinase kinase binding	0.01265	*Arrb1*; *Ksr1*
cysteine-type endopeptidase regulator activity involved in apoptotic process	0.046686	*Arrb1*; *Bad*

## Validation of RNA-seq Results

Real-time PCR was performed on cDNA samples to validate the expression levels of 20 DEGs (10 up-regulated and 10 down-regulated genes) using real-time PCR. We validated the genes that met the following principles: firstly, the fold-change of DEGs was as large as possible. Secondly, the gene expression, at the transcription level, was enough to be detected *via* real-time PCR. Thirdly, the genes were associated with different biological features between W.ISCs and GK.ISCs, such as proliferation, migration, and ECM components synthesis.

**Figure 4:**
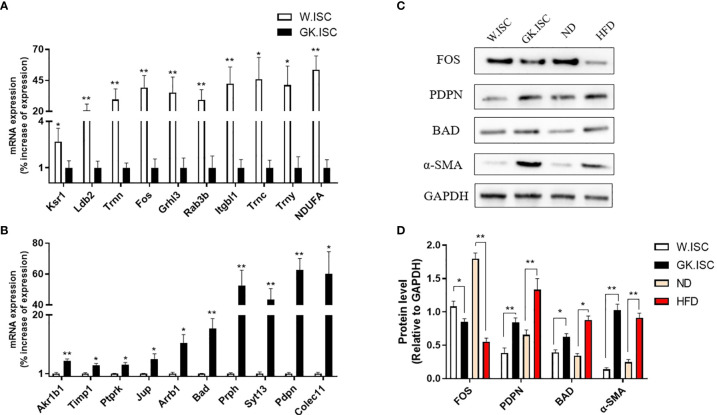
Validation of RNA-seq. **(A)** To validate the 10 differential expression genes which over-expresses in Wistar ISCs by real-time PCR. Wistar ISCs compared with GK ISCs. **(B)** To validate the 10 differential expression genes which over-expressed in GK ISCs by real-time PCR. GK ISCs compared with Wistar ISCs. **(C)** Western blotting of ISCs by PDPN, FOS, Bad and α-SMA antibody in Wistar rat, GK rat, normal diet SD rat (ND) and high-fat diet SD rat (HFD). **(D)** Statistical analysis of the Western blotting. Data are expressed as means ± SEM (n = 3), *P < 0.05, **P < 0.01.

The results of real-time PCR confirmed the analogous differential regulation of these genes in ISCs to that of the mRNA sequence dataset. Hence, the gene expression patterns in the sequencing data were consistent with the results obtained by real-time PCR (**Figures 4A, B**, β-actin as the reference gene; **Figure S2**, 18s rRNA as the reference gene). Combined with the real-time PCR and functional analysis of DEGs, we performed western blotting experiments to confirm the protein expression levels of Podoplanin, FOS and Bad in GK.ISCs, HFD SD rat ISCs, and their controls. As shown in **Figures 4C, D**, the expression level of FOS was significantly reduced compared with controls (GK.ISC *vs* W.ISC: *P* < 0.05; HFD *vs* ND: *P* < 0.01), podoplanin (GK.ISC *vs* W.ISC: *P* < 0.01; HFD *vs* ND: *P* < 0.01) and Bad (GK.ISC *vs* W.ISC: *P* < 0.05; HFD *vs* ND: *P* < 0.05) were significantly increased in GK.ISCs, which were consistent with the expression of α-SMA.

Immunofluorescence observed a less FOS expression but a more abundant expression of podoplanin and BAD in HFD rat group compared with ND rat group. These were consistent with the results from western blotting (**Figure S3**).

## Discussion

Islet fibrosis has been observed in the advanced stage of T2DM islets, which contributes to the progression of β cell dysfunction in diabetic patients and animal models (3, 4, 24–27). The pathological feature of islets fibrosis is the deposition of ECM components, including Collagen-I (Col-I), Collagen-III (Col-III), and fibronectin. However, the mechanisms underlying islet fibrosis remain largely unknown, though it is reported that the inflammatory cytokine, amyloid deposition, activation of RAS, and oxidative stress might be involved in (10, 28). We previously found that ISCs are responsible for islet fibrosis (14). However, we also demonstrated that ISCs and PSCs are similar but not identical *via* transcriptome sequencing (17). Compared with PSCs, ISCs exhibit lower proliferation and migration rates, are more easily to be activated by stimulators, and have fewer lipid droplets. In T2DM, ISCs transform from a quiescent into an activated state. ISCs express a large amount of α-SMA and exhibit increased proliferation and migration rates, as well as the enhanced synthesis and deposition of ECM components during activation, which are associated with fibrogenic response (21, 29). Therefore, it is necessary to understand the mechanisms leading to the activation of ISCs in order to identify potential targets to prevent the progression of islet fibrosis in T2DM.

In this study we performed an RNA-sequencing on cultured GK rats’ ISCs and Wistar rats’ ISCs to unravel the potential key genes involved in diabetes-induced activation of ISCs. The results showed 204 differentially expressed genes in transcription levels of GK.ISCs and W.ISCs, accounting for 0.58% of all the 35,362 genes detected. After functional analyses of these differentially expressed genes, along with GO and KEGG enrichment analyses, the mRNA levels of these genes were further confirmed by real-time PCR in cultured ISCs. Among the selected genes for validation in real-time PCR, the gene *Pdpn*, *Fos*, *Bad*, *Akr1b1*, *Timp1*, *Ptprk*, *Jup* were involved in cell proliferation and cell migration GO terms. And *Arrb1* and *Ksr1* were involved in mitogen-activated protein kinase binding and cysteine-type endopeptidase regulator activity involved in the apoptotic process, which may participate in cell proliferation, differentiation, transformation, and apoptosis in activated ISCs. Additionally, *Trnc*, *Trny* and *Trnn* were involved in Aminoacyl-tRNA biosynthesis KEGG pathway, which may contribute to the differences in protein synthesis between GK.ISCs and W.ISCs.

We selected *Fos*, *Pdpn, and Bad* as the potential key genes for diabetes-induced activation of ISCs based on the results of real-time PCR and functional analyses. And we then tested the protein expression levels of FOS, Podoplanin, and Bad by western blotting and immunofluorescence. The results showed that the expression level of FOS was significantly decreased, and podoplanin and Bad were significantly increased in GK.ISCs compared with controls, which were consistent with the expression of α-SMA.

The *Fos* proto-oncogene, belonging to the *Fos* gene family, can be rapidly and transiently induced in a wide range of cell types in response to growth factors, cytokines, serum, and other extracellular stimuli. The influence of *Fos* on stellate cells has been reported in several studies. Wei et al. showed that methyl helicterate treatment in hepatic stellate cells (HSCs) inhibited the expression of *Fos*, a downstream nuclear transcription factor of extracellular regulated protein kinases1/2 (ERK1/2) signaling pathway, and participated in many physiological processes (30). Gao et al. showed that the transient induction of *Fos* gene activation and expression was involved in connective tissue growth factor (CCN2)-stimulated HSCs DNA synthesis and cell proliferation *via* activating ERK1/2 signaling pathway (31). It indicates that *Fos* may contribute to the activation of HSCs and the progress of liver fibrosis. However, another protein in the *Fos* family, *Fosl1*, has shown to be a negative regulator of Col-I in PSCs (32). In lung epithelial cells, *Fosl1* also acts as a transcriptional repressor of Col-I (33). These findings are consistent with our results that the expression level of *Fos* is decreased in GK.ISCs. This difference in *Fos* regulation might be possibly due to the differences in genetic mutations or the differences between liver and pancreas.

Podoplanin, encoded by *Pdpn* gene, a mucin-type transmembrane sialoglycoprotein, is highly expressed in GK.ISCs (*vs* W.ISCs, adjust *P* < 0.01) detected from the RNA-seq. GO functional analysis shows that *Pdpn* plays important roles in cell proliferation, migration, pseudopod formation, cell activity, regulating tissue and organ development. These biological effects are closely related to the phenotype of ISCs in diabetes. In pathology conditions, a growing body of evidence indicates that podoplanin plays an important role in tumorigenesis (34). It has been proved that podoplanin has an influence on tumor-associated lymphangiogenesis and activation of cancer-associated fibroblasts (CAFs) (35–37). Podoplanin in CAFs is up-regulated in a variety of tumors, such as breast cancer, lung cancer, and pancreatic cancer. In particular, the CAFs of pancreatic ductal cancer are activated PSCs (38, 39). Podoplanin enhances the α-SMA expression, promotes the activation and proliferation of PSCs, and accelerates the migration and invasion of tumor cells. CAFs with high expression of podoplanin can also secrete a large number of cytokines and chemokines, such as TGF-β, tumor necrosis factor-α (TNF-α), interleukin-8 (IL-8), vascular endothelial growth factor (VEGF), which may promote tumor progression (38, 40).

Bcl-2 associated death promoter (Bad) was reported as a Bcl-2-binding protein in 1995 by Yang et al. (41). Bad is a promoter of cell apoptosis and has been shown to form dimers with the anti-apoptotic proteins Bcl-XL and Bcl-2. The pro-apoptotic action can be blocked by phosphorylation and binding with the 14-3-3 protein (42). It has been demonstrated that the binding of Bad to 14-3-3τ protein, a result of insulin-like growth factor I (IGF-I) induced Akt activation and phosphorylation of Ser 473 in activated HSCs, inhibited Bad binding to Bcl-XL and Bcl-2 (43). This indicates that p-Bad could protect HSCs from apoptosis and participate in the pro-fibrogenic process. In this study we observed that Bad is over-expressed in GK and HFD SD rats ISCs. Furthermore, Bad is involved in cell proliferation, migration and apoptosis after GO analysis. We, therefore, speculate that Bad or p-Bad could modulate apoptosis and cell growth in ISCs, and then participate in the fibrogenic process.

Although our study provides the estimates of the transcriptional differences between quiescent and activated ISCs, it has several limitations. First, GK rat is developed by repeated inbreeding of glucose-intolerant Wistar rat. Due to the accumulation of multiple mutations in GK rats, there are genomic differences between the two closely related rat strains. Thus, we detected the expression levels of 20 genes from the DEGs in HFD SD rats in order to minimize the bias. The results of real-time PCR and western blotting in HFD SD rats are consistent with those in GK rats, showing that the differences of transcriptome in GK.ISCs may commonly exist in diabetes-induced activated ISCs. Secondly, our study only validated the expression of potential key genes involved in diabetes-induced activation of ISCs, while we did not perform the functional experiments.

## Conclusion

In summary, this study is the first to describe the RNA-seq from the diabetes-induced activated ISCs, where a total of 204 DEGs were found between GK.ISCs and W.ISCs. Upon the validation of the expression of potential key genes from GK and HFD rats, it seems possible that differences in gene expression levels commonly exist in diabetes-induced activation of ISCs. Future studies that perform functional experiments on *Fos*, *Pdpn*, and *Bad* might be of importance to confirm their involvement in diabetes-induced activation of ISCs.

## Data Availability Statement

The raw data supporting the conclusions of this article will be made available by the authors, without undue reservation.

## Ethics Statement

The animal study was reviewed and approved by Southeast University Animal Care and Use Committee.

## Author Contributions

ZS and SQ conceived the study and participated in its design. XW and JW performed the RNA-seq and drafted the manuscript. YC, VC, and CN participated in the statistical analysis. LL, TL, QW, YY, SQ and VC participated in the language editing of the manuscript. All authors contributed to the article and approved the submitted version.

## Funding

The work was supported by the National Nature Science Foundation of China (NSFC-81870534) and partly by the Guangdong Basic and Applied Basic Research Foundation (Grant No. 2020A1515111021).

## Conflict of Interest

The authors declare that the research was conducted in the absence of any commercial or financial relationships that could be construed as a potential conflict of interest.

## Publisher’s Note

All claims expressed in this article are solely those of the authors and do not necessarily represent those of their affiliated organizations, or those of the publisher, the editors and the reviewers. Any product that may be evaluated in this article, or claim that may be made by its manufacturer, is not guaranteed or endorsed by the publisher.
