# Supplementary material for: Screening and Identification of Key Genes for Activation of Islet Stellate Cell
Source: Front Endocrinol (Lausanne). 2021 Sep 9;12:695467. doi: 10.3389/fendo.2021.695467 (PMC8458934; doi:10.3389/fendo.2021.695467)
Supplement: Supplementary file 1 [file DataSheet_1.docx]

***mRNA library construction and sequencing***

As described previously[25], total RNA was extracted from ISCs by the Trizol reagent (Invitrogen, CA, USA) according to the manufacturer’s protocol. The quantity and purity of the total RNA were detected via RNA 6000 Nano LabChip Kit (Agilent, CA, USA) and Bio analyzer 2200 with RIN more than 7.0. Then approximately 2 µg of total RNA were subjected to isolate the PolyA mRNA via polyT oligo-attached magnetic beads (Invitrogen). After purification, the mRNA was firstly fragmented into small pieces and then reverse-transcribed to create the final cDNA library based on the protocol of the mRNA-Seq sample preparation kit (Illumina, San Diego, USA). The average insert size for the paired-end libraries was about 300 bps. We then performed the paired-end sequencing (100 bps) using the Hiseq3000 platform.

***Functional enrichment analysis***

The sequenced raw data were filtered to remove low-quality tags such as reads with unknown nucleotides “N”, empty reads, and reads mapped at multiple sites in the reference genome. Then we matched the clean reads to the sequences in the Rattus genome database by Tophat (version 2.0.4) allowing up to two base mismatches. The mapped clean reads were regarded as precise clean reads. For two-factor analysis of variance, we calculated and normalized the number of unambiguous clean reads for each gene to log-counts per million using the limma package in R program.

All of the DEGs were used for the GO and KEGG enrichment analyses. For the GO analysis, a corrected P-value of <0.05 was considered as the threshold to determine significant enrichment of the gene sets. Same as GO analysis, a Q-value ≤0.05 was considered as the threshold to determine significant enrichment of the gene sets for KEGG enrichment analysis. The KOBAS v2.0 software was used for GO and KEGG analysis.
