# Supplementary material for: Screening and Identification of Key Genes for Activation of Islet Stellate Cell
Source: Front Endocrinol (Lausanne). 2021 Sep 9;12:695467. doi: 10.3389/fendo.2021.695467 (PMC8458934; doi:10.3389/fendo.2021.695467)
Supplement: Supplementary file 2 [file Table_1.docx]

Table S1 Sequences of primers for the target genes

| Gene | Forward Primer | Reverse Primer |
| --- | --- | --- |
| Ksr1 | GAAGGCAGATGCAAGATGCG | GGGGATCTCCTGTACCACCT |
| Ldb2 | CAGCAAGAAGAAGACCACA | CAAACTCACCTCCCATCA |
| Trnn | AGGCGGGAGAAGCCTTAGTA | AGATAGAAGACACCCCGGCT |
| Fos | TACTACCATTCCCCAGCCGA | GCGTATCTGTCAGCTCCCTC |
| Grhl3 | CCCCAGGTCCAAGTAAGCTG | GGGTCGTCTTTGAAGGTGCT |
| Itgbl1 | TCAGCAGCAATTCGACCACT | GACTAGCTTGAGTCAGGGCG |
| Trnc | GGACTAGCCCCCTTCCACTA | TACTAAGGCTTCTCCCGCCT |
| Trny | TGGTAAAAAGGGGCCTCAACC | ATGGCTGAGTAAGCATTAGACTG |
| NDUFA | CGGACCATCTGGGGAACAAA | TGCTATGACTTCAGGGCAGG |
| Akr1b1 | GAGCTGTGCCAAACACAAGG | TGACCCCCATAGGACTGGAG |
| Timp1 | CCTAGAGACACGCTAGAGCAG | CCAGGTCCGAGTTGCAGAAA |
| Ptprk | GCCACACTTTCAACGTCACC | CAACGTGTTGAGGGGCTTTG |
| Jup | ACGCTCAGGTACACCGGATA | GTGGATGCCCGAGTCATAGG |
| Arrb1 | TTGTGTTTGAGGACTTTGCT | CTATCTGTTGTTGAGGTGTGG |
| Bad | CTTGAGGAAGTCCGATCCCG | ACGTCAGCTCTTCTCCCTTG |
| Prph2 | GAGTAATGTGGATGGGAGGT | CGCAGAGTTGTTGGTGAG |
| Syt13 | GAAGACCACAGCAAAGGAG | TAATCAGCACCACCAGGA |
| Colec11 | GGCTTTCCTGTCCCTACT | CTTTGTTTCCCTTTTCTCCT |
| Pdpn | CATTGAGGAACTGCCGACCT | TTTCACCGCCTGCGTTATCT |
| Rab3b | CATCACCACAGCCTACTACC | ATCACCCTTTCCTCTTCC |
| β-actin | GAGAGGGAAATCGTGCGTGACA | ACCCAAGAAGGAAGGCTGGAAA |
| 18s rRNA | GTAACCCGTTGAACCCCATT | CCATCCAATCGGTAGTAGCG |
