# Supplementary material for: Screening and Identification of Key Genes for Activation of Islet Stellate Cell
Source: Front Endocrinol (Lausanne). 2021 Sep 9;12:695467. doi: 10.3389/fendo.2021.695467 (PMC8458934; doi:10.3389/fendo.2021.695467)
Supplement: Supplementary file 3 [file Table_2.docx]

Table S2 The DEGs detected in the RNA-seq

| Gene ID | A12 | B12 | C12 | D12 | E12 | F12 | foldChange | pval | padj |
| --- | --- | --- | --- | --- | --- | --- | --- | --- | --- |
| RGD1562420 | 0 | 0 | 0 | 1767 | 1668 | 2066 | Inf | 1.16E-65 | 2.56E-61 |
| RGD1565987 | 0 | 0 | 0 | 46 | 50 | 63 | Inf | 2.62E-08 | 1.48E-05 |
| Abcg3l2 | 0 | 0 | 0 | 18 | 45 | 25 | Inf | 3.06E-06 | 0.000804 |
| Prph2 | 0 | 0 | 0 | 15 | 45 | 23 | Inf | 4.64E-06 | 0.001138 |
| LOC100363221 | 0 | 0 | 0 | 13 | 19 | 23 | Inf | 6.17E-05 | 0.010394 |
| LOC305639 | 0 | 0 | 0 | 16 | 25 | 8 | Inf | 0.000128 | 0.018702 |
| Akr1b10 | 0 | 0 | 0 | 6 | 21 | 15 | Inf | 0.000321 | 0.037294 |
| LOC102552640 | 0 | 0 | 0 | 33 | 10 | 72 | Inf | 0.000423 | 0.046627 |
| LOC103690108 | 2 | 2 | 1 | 1563 | 2481 | 2225 | 1183.511 | 7.15E-64 | 5.26E-60 |
| Flg | 2 | 0 | 0 | 8 | 174 | 161 | 149.5103 | 0.000178 | 0.023874 |
| LOC678760 | 3 | 3 | 5 | 467 | 571 | 625 | 146.1127 | 1.66E-26 | 4.07E-23 |
| Sez6l | 1 | 0 | 2 | 65 | 115 | 62 | 76.47953 | 3.45E-08 | 1.85E-05 |
| RT1-A1 | 46 | 41 | 52 | 1706 | 3274 | 5472 | 71.91078 | 1.31E-07 | 5.35E-05 |
| Syt13 | 8 | 9 | 37 | 875 | 1148 | 577 | 46.76004 | 4.49E-23 | 9.01E-20 |
| Bnc1 | 0 | 4 | 1 | 99 | 30 | 69 | 40.50675 | 4.18E-05 | 0.007679 |
| Colec11 | 0 | 3 | 2 | 37 | 74 | 89 | 39.01574 | 1.30E-06 | 0.000398 |
| LOC100359701 | 0 | 0 | 3 | 31 | 28 | 42 | 33.93137 | 5.27E-05 | 0.009304 |
| Celsr2 | 16 | 27 | 15 | 942 | 515 | 341 | 30.60292 | 3.12E-05 | 0.005927 |
| Upk1b | 8 | 40 | 41 | 969 | 321 | 1235 | 28.81572 | 3.49E-05 | 0.006557 |
| LOC100360828 | 10 | 2 | 1 | 104 | 153 | 115 | 26.2334 | 8.36E-08 | 3.69E-05 |
| RGD1565779 | 0 | 2 | 4 | 46 | 15 | 61 | 20.87884 | 0.000437 | 0.047652 |
| LOC103691175 | 2 | 0 | 2 | 23 | 39 | 26 | 20.61315 | 0.000277 | 0.033793 |
| LOC100363472 | 0 | 3 | 3 | 44 | 42 | 33 | 19.69241 | 0.000136 | 0.019538 |
| LOC690581 | 6 | 3 | 6 | 73 | 155 | 65 | 18.17579 | 3.72E-06 | 0.000953 |
| Krt7 | 840 | 505 | 954 | 11051 | 9662 | 19624 | 17.05007 | 1.89E-07 | 7.45E-05 |
| LOC102552147 | 18 | 10 | 23 | 348 | 200 | 249 | 15.36725 | 1.34E-08 | 7.97E-06 |
| LOC103693757 | 134 | 144 | 136 | 2675 | 1047 | 2310 | 14.47717 | 1.59E-05 | 0.003399 |
| LOC102547438 | 3 | 2 | 6 | 36 | 63 | 50 | 12.93746 | 0.000275 | 0.033628 |
| Ctsw | 5 | 3 | 2 | 33 | 60 | 40 | 12.36787 | 0.000405 | 0.0449 |
| LOC100911555 | 19 | 16 | 14 | 141 | 348 | 148 | 12.02825 | 2.86E-06 | 0.00076 |
| Ablim3 | 10 | 18 | 16 | 181 | 209 | 123 | 11.25164 | 2.26E-06 | 0.000614 |
| LOC102555453 | 0 | 4 | 16 | 72 | 88 | 66 | 11.18775 | 0.000162 | 0.022476 |
| Asb2 | 6 | 6 | 12 | 69 | 140 | 40 | 9.758206 | 0.00026 | 0.032439 |
| Bad | 101 | 159 | 126 | 940 | 1292 | 1674 | 9.752217 | 2.84E-12 | 2.72E-09 |
| Cxcr7 | 361 | 483 | 550 | 5615 | 2951 | 5077 | 9.708367 | 7.30E-07 | 0.000244 |
| Adra1d | 19 | 30 | 13 | 227 | 322 | 84 | 9.638649 | 0.000176 | 0.02384 |
| Tbkbp1 | 19 | 29 | 30 | 241 | 311 | 215 | 9.457125 | 4.33E-07 | 0.000149 |
| Il18rap | 18 | 8 | 20 | 98 | 169 | 163 | 8.856968 | 3.02E-05 | 0.005792 |
| Abcg1 | 6 | 12 | 16 | 57 | 141 | 90 | 8.038836 | 0.000313 | 0.036736 |
| Cotl1 | 808 | 670 | 1224 | 5000 | 6827 | 9732 | 7.69543 | 7.35E-08 | 3.36E-05 |
| Rtn4r | 18 | 50 | 39 | 153 | 369 | 324 | 7.538172 | 1.65E-06 | 0.000479 |
| Trns1 | 922 | 1037 | 977 | 7230 | 9271 | 5698 | 7.220544 | 2.06E-17 | 3.03E-14 |
| Metrnl | 591 | 491 | 646 | 3046 | 3820 | 5835 | 7.074666 | 4.80E-07 | 0.000163 |
| Gprin2 | 15 | 12 | 23 | 126 | 68 | 154 | 6.885331 | 0.000398 | 0.044495 |
| Pdpn | 92 | 167 | 309 | 980 | 1051 | 1913 | 6.832027 | 4.49E-05 | 0.008178 |
| Tdrp | 83 | 151 | 163 | 810 | 604 | 1246 | 6.613232 | 3.00E-05 | 0.005792 |
| LOC678766 | 165 | 180 | 240 | 1343 | 912 | 1455 | 6.235565 | 3.84E-08 | 2.02E-05 |
| Tuba4a | 185 | 262 | 437 | 1030 | 1789 | 2711 | 6.053389 | 0.000327 | 0.037578 |
| Plcxd2 | 52 | 50 | 48 | 297 | 361 | 204 | 5.47997 | 2.44E-05 | 0.004846 |
| Anxa3 | 636 | 482 | 1157 | 3847 | 3616 | 3960 | 4.892348 | 2.29E-11 | 1.81E-08 |
| LOC100361993 | 345 | 341 | 402 | 1080 | 1722 | 2573 | 4.737721 | 0.000282 | 0.033969 |
| Fxyd6 | 770 | 1433 | 1141 | 5430 | 6831 | 3862 | 4.634403 | 3.54E-11 | 2.69E-08 |
| Grem2 | 3124 | 2471 | 3441 | 8756 | 16271 | 18907 | 4.622207 | 5.48E-06 | 0.001328 |
| LOC100911717 | 88 | 103 | 95 | 408 | 318 | 625 | 4.615156 | 0.000118 | 0.017533 |
| Kcng1 | 306 | 100 | 469 | 967 | 1696 | 1124 | 4.099393 | 2.03E-07 | 7.71E-05 |
| Ccdc85c | 78 | 100 | 97 | 312 | 386 | 448 | 4.017554 | 0.000114 | 0.017047 |
| Hebp2 | 156 | 172 | 184 | 560 | 541 | 879 | 3.757093 | 2.34E-05 | 0.004817 |
| Wfdc1 | 1368 | 1986 | 1555 | 6393 | 8969 | 3559 | 3.665075 | 0.000188 | 0.024649 |
| Arrb1 | 810 | 470 | 1068 | 2167 | 3845 | 2403 | 3.387102 | 1.98E-07 | 7.65E-05 |
| Ttc9 | 313 | 334 | 299 | 865 | 1522 | 797 | 3.170447 | 2.36E-05 | 0.004821 |
| LOC100910984 | 143 | 221 | 154 | 548 | 551 | 571 | 3.122276 | 0.000366 | 0.04139 |
| Ank3 | 203 | 151 | 402 | 855 | 902 | 663 | 3.103601 | 0.000118 | 0.017533 |
| LOC681584 | 1047 | 1178 | 1186 | 2424 | 3819 | 4803 | 3.100455 | 6.40E-05 | 0.010695 |
| Taf13 | 349 | 459 | 470 | 985 | 1438 | 1300 | 2.79149 | 0.000104 | 0.015744 |
| Syndig1 | 596 | 568 | 705 | 1210 | 2226 | 1896 | 2.705814 | 5.08E-05 | 0.009028 |
| Jup | 1424 | 1826 | 1271 | 3633 | 5781 | 3219 | 2.643993 | 1.23E-05 | 0.002757 |
| Dock8 | 389 | 374 | 526 | 720 | 1521 | 1289 | 2.593099 | 0.00029 | 0.034536 |
| Ptgr1 | 753 | 606 | 697 | 1311 | 2362 | 1924 | 2.570796 | 9.29E-05 | 0.014422 |
| Hip1r | 443 | 372 | 471 | 777 | 1173 | 1493 | 2.559838 | 0.000402 | 0.044777 |
| Pcbp3 | 602 | 582 | 914 | 1599 | 1638 | 2227 | 2.530187 | 0.000186 | 0.024602 |
| Ptprk | 760 | 614 | 842 | 1753 | 2032 | 1955 | 2.487699 | 0.000183 | 0.024271 |
| Timp1 | 3149 | 3139 | 3303 | 5673 | 8408 | 9566 | 2.357086 | 9.27E-05 | 0.014422 |
| Akr1b1 | 3468 | 3472 | 4541 | 9276 | 7362 | 10880 | 2.341569 | 0.000158 | 0.022404 |
| Acvrl1 | 3317 | 3513 | 3562 | 821 | 1873 | 2202 | 0.446679 | 0.000425 | 0.046627 |
| Raph1 | 9500 | 9773 | 9906 | 2630 | 6148 | 5054 | 0.446203 | 0.000359 | 0.04076 |
| S1pr3 | 5259 | 4629 | 4050 | 2769 | 2390 | 1220 | 0.440631 | 0.00021 | 0.027258 |
| Adcy5 | 2174 | 2161 | 2204 | 959 | 1117 | 807 | 0.422404 | 0.000281 | 0.033969 |
| Prss35 | 3891 | 5211 | 3442 | 2512 | 1041 | 1768 | 0.421152 | 0.000335 | 0.038259 |
| Fzd4 | 1468 | 1397 | 2099 | 941 | 624 | 561 | 0.4206 | 0.000316 | 0.03682 |
| Ssc5d | 3938 | 5183 | 4032 | 2542 | 1911 | 1196 | 0.418521 | 7.17E-05 | 0.011622 |
| Lhfp | 12044 | 13962 | 12466 | 2880 | 7012 | 6931 | 0.412829 | 0.000104 | 0.015741 |
| Tbxa2r | 2072 | 2019 | 1880 | 1199 | 923 | 397 | 0.408961 | 0.000169 | 0.023142 |
| Pkig | 7215 | 9181 | 7148 | 1942 | 5097 | 3063 | 0.400962 | 5.69E-05 | 0.009802 |
| Hspb2 | 2042 | 1986 | 1810 | 968 | 501 | 861 | 0.392762 | 7.37E-05 | 0.01186 |
| Psd3 | 2185 | 2168 | 1867 | 912 | 699 | 885 | 0.390056 | 6.97E-05 | 0.011392 |
| Ifit2 | 1818 | 2421 | 2378 | 1188 | 1096 | 323 | 0.38104 | 4.52E-05 | 0.008178 |
| Pkdcc | 888 | 1182 | 910 | 488 | 388 | 250 | 0.367492 | 0.000211 | 0.027258 |
| Ehbp1 | 2198 | 3042 | 2377 | 943 | 894 | 1044 | 0.366987 | 1.61E-05 | 0.003423 |
| Egr1 | 11591 | 12380 | 12149 | 7345 | 2121 | 3705 | 0.365972 | 2.86E-06 | 0.00076 |
| Synpo2 | 5864 | 4670 | 5700 | 1635 | 3341 | 1306 | 0.360958 | 8.47E-06 | 0.001965 |
| Rps6ka5 | 794 | 1015 | 810 | 254 | 391 | 334 | 0.356234 | 0.000273 | 0.033628 |
| Wscd2 | 1234 | 1600 | 1242 | 601 | 840 | 32 | 0.34029 | 0.000124 | 0.018219 |
| Gas1 | 1237 | 1451 | 1289 | 318 | 685 | 417 | 0.335484 | 3.97E-05 | 0.007361 |
| Flot1 | 4000 | 4556 | 4630 | 1304 | 2127 | 1217 | 0.334085 | 1.54E-06 | 0.000453 |
| Unc5c | 1128 | 1087 | 737 | 242 | 168 | 585 | 0.328427 | 0.000222 | 0.028302 |
| Crip2 | 3930 | 3246 | 4266 | 440 | 1466 | 2034 | 0.325159 | 1.17E-06 | 0.000379 |
| LOC103694903 | 859 | 745 | 981 | 263 | 350 | 261 | 0.322866 | 7.91E-05 | 0.012633 |
| Ksr1 | 509 | 635 | 449 | 72 | 117 | 338 | 0.319331 | 0.000312 | 0.036736 |
| Gucy1a2 | 514 | 658 | 582 | 290 | 174 | 100 | 0.316324 | 0.000165 | 0.022704 |
| Spata13 | 711 | 930 | 765 | 155 | 429 | 229 | 0.315079 | 0.000103 | 0.015734 |
| Mylk | 32065 | 40614 | 36112 | 8208 | 21313 | 7357 | 0.314771 | 6.52E-06 | 0.001564 |
| Hrct1 | 2212 | 2168 | 1672 | 293 | 1053 | 712 | 0.314712 | 4.53E-06 | 0.001123 |
| Tspan18 | 2874 | 4196 | 2905 | 1964 | 853 | 300 | 0.311236 | 0.000172 | 0.023373 |
| LOC102554200 | 562 | 806 | 830 | 237 | 294 | 170 | 0.306375 | 6.81E-05 | 0.011213 |
| Klf2 | 4662 | 3936 | 4516 | 1581 | 1779 | 688 | 0.294478 | 8.69E-08 | 3.76E-05 |
| Kctd12 | 499 | 565 | 456 | 126 | 177 | 162 | 0.291918 | 0.000159 | 0.022404 |
| Bhlhe41 | 990 | 1284 | 1055 | 492 | 380 | 115 | 0.288265 | 4.47E-06 | 0.00112 |
| Trpc6 | 538 | 349 | 523 | 224 | 182 | 15 | 0.287672 | 0.00016 | 0.022404 |
| Col6a2 | 16011 | 21310 | 16720 | 11804 | 1085 | 1780 | 0.280556 | 0.000215 | 0.027602 |
| Cav2 | 1876 | 1811 | 1629 | 326 | 916 | 358 | 0.27794 | 8.00E-07 | 0.000263 |
| Stc2 | 280 | 373 | 318 | 127 | 34 | 105 | 0.275178 | 0.0003 | 0.03561 |
| St6galnac2 | 241 | 411 | 243 | 59 | 33 | 155 | 0.272467 | 0.000447 | 0.048445 |
| Ube2q2l | 2675 | 2077 | 2441 | 657 | 970 | 386 | 0.263859 | 5.57E-08 | 2.79E-05 |
| Egr2 | 399 | 584 | 406 | 266 | 64 | 25 | 0.259785 | 7.99E-05 | 0.012672 |
| Trabd2b | 678 | 783 | 611 | 159 | 153 | 240 | 0.258042 | 8.38E-06 | 0.001965 |
| Kcnd3 | 1005 | 1206 | 1070 | 374 | 113 | 341 | 0.252412 | 3.58E-07 | 0.000127 |
| Lrrc25 | 938 | 850 | 1127 | 20 | 86 | 649 | 0.25201 | 0.000134 | 0.019457 |
| Gdf15 | 1291 | 1190 | 1537 | 28 | 111 | 867 | 0.243739 | 5.45E-05 | 0.009544 |
| Islr | 2780 | 4162 | 2644 | 1114 | 1129 | 162 | 0.240551 | 1.34E-06 | 0.000405 |
| Stard8 | 721 | 867 | 729 | 82 | 88 | 388 | 0.234902 | 1.19E-06 | 0.000379 |
| Wisp2 | 12429 | 13222 | 10674 | 3984 | 3699 | 1012 | 0.229939 | 1.06E-10 | 7.53E-08 |
| Hey2 | 346 | 286 | 290 | 174 | 30 | 2 | 0.22818 | 5.80E-05 | 0.009913 |
| Megf10 | 227 | 351 | 275 | 112 | 17 | 56 | 0.221168 | 6.79E-05 | 0.011213 |
| Cdh13 | 4949 | 5721 | 3330 | 428 | 2657 | 378 | 0.220614 | 5.69E-05 | 0.009802 |
| Ppp1r14c | 365 | 410 | 294 | 70 | 118 | 46 | 0.205567 | 2.38E-05 | 0.004823 |
| Pde1a | 505 | 747 | 546 | 101 | 189 | 101 | 0.205216 | 1.24E-06 | 0.000385 |
| Sepp1 | 2089 | 3566 | 2065 | 280 | 707 | 678 | 0.203791 | 2.18E-06 | 0.000614 |
| LOC102556446 | 194 | 239 | 226 | 22 | 103 | 19 | 0.19804 | 0.000258 | 0.032374 |
| Galnt16 | 898 | 859 | 671 | 187 | 203 | 100 | 0.192598 | 6.30E-08 | 2.96E-05 |
| Vstm4 | 351 | 332 | 369 | 14 | 38 | 157 | 0.191851 | 9.69E-06 | 0.002225 |
| Col19a1 | 200 | 146 | 217 | 21 | 97 | 1 | 0.188506 | 0.000392 | 0.044071 |
| Tnik | 973 | 948 | 962 | 224 | 155 | 167 | 0.184898 | 6.68E-09 | 4.21E-06 |
| Akr1b8 | 715 | 626 | 853 | 84 | 228 | 107 | 0.177632 | 5.69E-08 | 2.79E-05 |
| Prps2 | 2339 | 4274 | 2694 | 688 | 292 | 619 | 0.171161 | 1.14E-07 | 4.73E-05 |
| Slc7a2 | 1366 | 2377 | 1146 | 129 | 139 | 580 | 0.169261 | 6.08E-05 | 0.010313 |
| Avil | 174 | 126 | 180 | 25 | 41 | 20 | 0.168822 | 0.000241 | 0.030356 |
| LOC102550180 | 898 | 725 | 436 | 38 | 130 | 191 | 0.163168 | 2.49E-05 | 0.004894 |
| Nalcn | 161 | 147 | 136 | 22 | 47 | 8 | 0.160197 | 0.000272 | 0.033628 |
| Akr1e2 | 220 | 311 | 223 | 18 | 47 | 61 | 0.158535 | 1.43E-05 | 0.003121 |
| Rasl11a | 407 | 346 | 353 | 62 | 99 | 25 | 0.157554 | 1.23E-06 | 0.000385 |
| RT1-A2 | 482 | 592 | 649 | 23 | 39 | 205 | 0.151245 | 1.57E-08 | 9.12E-06 |
| RGD1562690 | 279 | 269 | 316 | 31 | 37 | 62 | 0.145306 | 2.23E-06 | 0.000614 |
| RT1-N2 | 561 | 590 | 632 | 38 | 114 | 121 | 0.144255 | 1.02E-08 | 6.24E-06 |
| Htr2b | 612 | 828 | 593 | 87 | 190 | 30 | 0.139912 | 2.88E-09 | 1.87E-06 |
| Pitx2 | 90 | 125 | 72 | 0 | 2 | 39 | 0.139653 | 0.000448 | 0.048445 |
| Tnfsf18 | 515 | 1205 | 1043 | 77 | 85 | 230 | 0.13928 | 1.36E-06 | 0.000405 |
| Ramp1 | 363 | 393 | 441 | 31 | 144 | 9 | 0.138137 | 2.69E-07 | 9.88E-05 |
| Figf | 6619 | 10687 | 5716 | 1165 | 1080 | 1030 | 0.137806 | 6.31E-08 | 2.96E-05 |
| Ccdc152 | 143 | 191 | 122 | 7 | 37 | 24 | 0.137495 | 9.18E-05 | 0.014422 |
| Slc7a3 | 194 | 187 | 163 | 32 | 28 | 15 | 0.132915 | 2.04E-05 | 0.004284 |
| Enox1 | 115 | 126 | 117 | 8 | 33 | 9 | 0.127341 | 0.000179 | 0.023874 |
| Ldb2 | 505 | 643 | 498 | 84 | 85 | 48 | 0.126905 | 2.35E-09 | 1.57E-06 |
| Wwc1 | 187 | 200 | 143 | 23 | 10 | 34 | 0.124698 | 1.24E-05 | 0.00276 |
| Rerg | 66 | 144 | 73 | 2 | 6 | 26 | 0.116712 | 0.000193 | 0.025135 |
| Art4 | 144 | 175 | 82 | 3 | 9 | 35 | 0.112494 | 3.51E-05 | 0.006557 |
| Mettl2b | 644 | 618 | 661 | 63 | 74 | 80 | 0.108437 | 5.03E-11 | 3.70E-08 |
| Prss12 | 571 | 677 | 646 | 124 | 32 | 45 | 0.107145 | 2.21E-11 | 1.81E-08 |
| Notch3 | 4245 | 4687 | 1888 | 842 | 247 | 26 | 0.103202 | 1.07E-05 | 0.002428 |
| Dpep2 | 608 | 571 | 678 | 73 | 42 | 72 | 0.099065 | 1.22E-11 | 1.03E-08 |
| Inpp4b | 2883 | 1888 | 2128 | 177 | 475 | 81 | 0.096908 | 6.20E-12 | 5.70E-09 |
| LOC103694585 | 1017 | 926 | 1137 | 78 | 169 | 64 | 0.094223 | 3.16E-14 | 3.67E-11 |
| Fos | 2757 | 2426 | 3160 | 423 | 107 | 239 | 0.092722 | 7.66E-21 | 1.41E-17 |
| Lgals9 | 190 | 227 | 292 | 3 | 8 | 56 | 0.092421 | 1.47E-07 | 5.91E-05 |
| Trna | 636 | 640 | 863 | 64 | 100 | 43 | 0.091732 | 1.33E-12 | 1.34E-09 |
| Sgcg | 1506 | 1680 | 1992 | 226 | 159 | 56 | 0.083303 | 5.50E-19 | 8.66E-16 |
| Srm | 207 | 260 | 205 | 19 | 23 | 12 | 0.076798 | 5.61E-08 | 2.79E-05 |
| Rftn2 | 99 | 93 | 84 | 14 | 4 | 2 | 0.072845 | 1.42E-05 | 0.003121 |
| Art3 | 279 | 407 | 149 | 0 | 0 | 62 | 0.072837 | 8.35E-06 | 0.001965 |
| F2rl2 | 84 | 90 | 75 | 8 | 2 | 8 | 0.072298 | 2.29E-05 | 0.004763 |
| LOC102553699 | 115 | 110 | 84 | 5 | 11 | 4 | 0.060041 | 4.28E-06 | 0.001086 |
| Rpl10l | 185 | 161 | 148 | 2 | 0 | 28 | 0.05981 | 9.55E-08 | 4.05E-05 |
| Bmp3 | 1455 | 2189 | 2048 | 114 | 196 | 40 | 0.057888 | 2.68E-23 | 5.90E-20 |
| Ar | 179 | 200 | 75 | 0 | 4 | 23 | 0.056949 | 1.87E-06 | 0.000535 |
| Slit3 | 5532 | 13456 | 4008 | 599 | 234 | 285 | 0.048567 | 0.000284 | 0.034041 |
| Fam181b | 129 | 137 | 78 | 4 | 2 | 10 | 0.045606 | 3.23E-07 | 0.000117 |
| Ephx2 | 121 | 188 | 127 | 2 | 13 | 4 | 0.039534 | 3.26E-08 | 1.80E-05 |
| Grid2 | 23 | 90 | 51 | 1 | 3 | 2 | 0.034581 | 9.63E-05 | 0.014849 |
| Mst1 | 454 | 498 | 433 | 1 | 5 | 40 | 0.032268 | 9.65E-17 | 1.33E-13 |
| Palmd | 1120 | 2383 | 1556 | 4 | 11 | 127 | 0.027613 | 2.06E-13 | 2.27E-10 |
| LOC103691813 | 44 | 37 | 46 | 0 | 4 | 0 | 0.026963 | 1.54E-05 | 0.003324 |
| Naalad2 | 15 | 39 | 15 | 0 | 2 | 0 | 0.024989 | 0.000161 | 0.022404 |
| Trnn | 197 | 188 | 233 | 3 | 9 | 3 | 0.022409 | 8.23E-12 | 7.26E-09 |
| Fam124a | 57 | 123 | 84 | 3 | 3 | 0 | 0.021901 | 7.46E-08 | 3.36E-05 |
| LOC103692171 | 76 | 60 | 102 | 0 | 6 | 0 | 0.021652 | 2.39E-07 | 8.92E-05 |
| LOC100364958 | 221 | 303 | 300 | 9 | 7 | 1 | 0.020095 | 1.70E-14 | 2.08E-11 |
| LOC100364769 | 52 | 50 | 76 | 2 | 1 | 0 | 0.016739 | 4.02E-07 | 0.000141 |
| Epc2l1 | 39 | 29 | 61 | 2 | 0 | 0 | 0.016427 | 2.22E-06 | 0.000614 |
| Rbm20 | 738 | 229 | 256 | 1 | 9 | 11 | 0.01566 | 0.000239 | 0.030315 |
| LOC102554737 | 28 | 15 | 27 | 1 | 0 | 0 | 0.015002 | 4.64E-05 | 0.008317 |
| Fras1 | 68 | 189 | 48 | 3 | 0 | 1 | 0.013665 | 0.000153 | 0.02189 |
| Itgbl1 | 5658 | 7149 | 4791 | 17 | 214 | 34 | 0.013331 | 5.15E-46 | 2.84E-42 |
| LOC681355 | 195 | 127 | 210 | 6 | 0 | 0 | 0.011875 | 6.25E-13 | 6.56E-10 |
| Grhl3 | 11 | 47 | 63 | 1 | 0 | 0 | 0.008907 | 2.86E-05 | 0.005575 |
| LOC100362069 | 1006 | 983 | 964 | 10 | 5 | 11 | 0.008679 | 1.23E-34 | 5.42E-31 |
| LOC103692946 | 681 | 636 | 771 | 2 | 9 | 8 | 0.008481 | 3.64E-29 | 1.00E-25 |
| RGD1564247 | 95 | 76 | 88 | 0 | 2 | 0 | 0.006598 | 2.16E-09 | 1.49E-06 |
| Rab3b | 638 | 367 | 775 | 3 | 5 | 4 | 0.006394 | 1.54E-19 | 2.61E-16 |
| Trnc | 541 | 532 | 698 | 4 | 4 | 1 | 0.004887 | 1.28E-29 | 4.04E-26 |
| RGD1359290 | 3670 | 4232 | 3919 | 19 | 11 | 28 | 0.00483 | 6.81E-65 | 7.51E-61 |
| Ndufa10l1 | 495 | 497 | 516 | 1 | 0 | 2 | 0.002005 | 5.56E-30 | 2.04E-26 |
| Trny | 151 | 169 | 207 | 0 | 0 | 0 | 0 | 1.36E-16 | 1.76E-13 |
| Rpl39l | 36 | 38 | 10 | 0 | 0 | 0 | 0 | 3.45E-06 | 0.000894 |
| Cntnap5c | 17 | 21 | 22 | 0 | 0 | 0 | 0 | 2.43E-05 | 0.004846 |
| Mettl24 | 11 | 20 | 8 | 0 | 0 | 0 | 0 | 0.000323 | 0.037335 |
