# Supplementary figures and images for: Screening and Identification of Key Genes for Activation of Islet Stellate Cell
Source: Front Endocrinol (Lausanne). 2021 Sep 9;12:695467. doi: 10.3389/fendo.2021.695467 (PMC8458934; doi:10.3389/fendo.2021.695467)

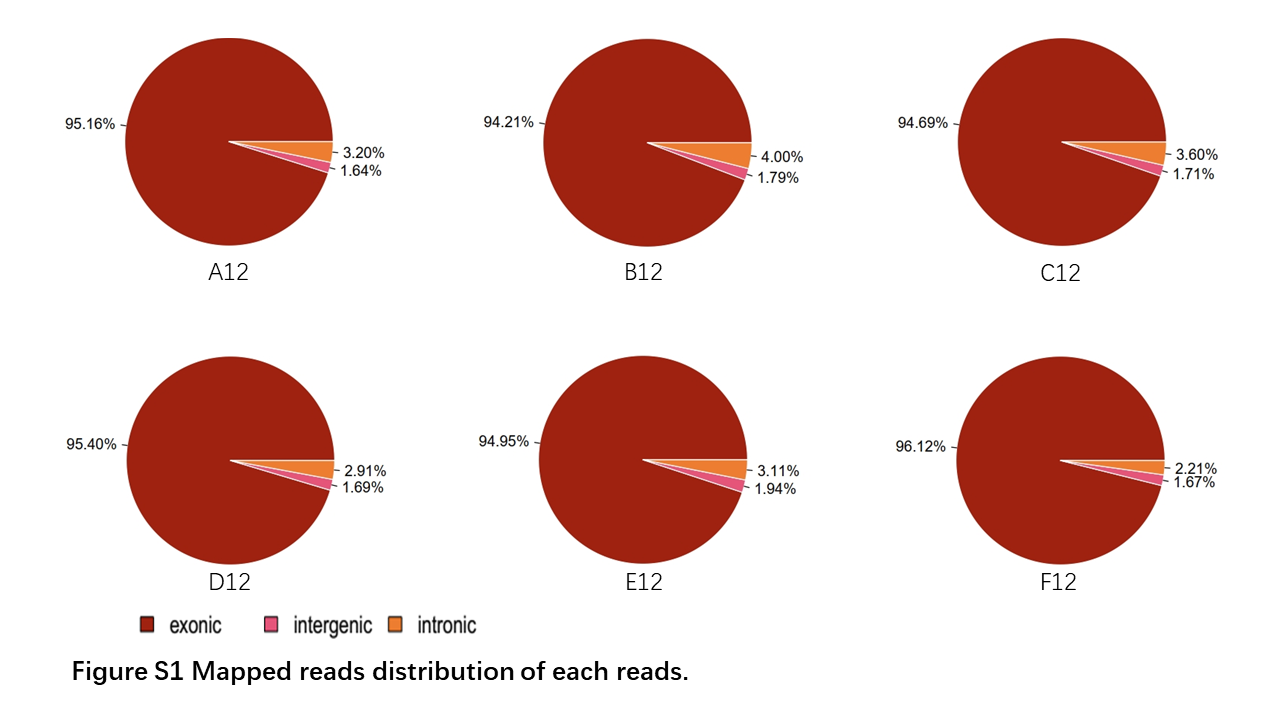

Supplement: Supplementary file 4 [file Image_1.jpeg]

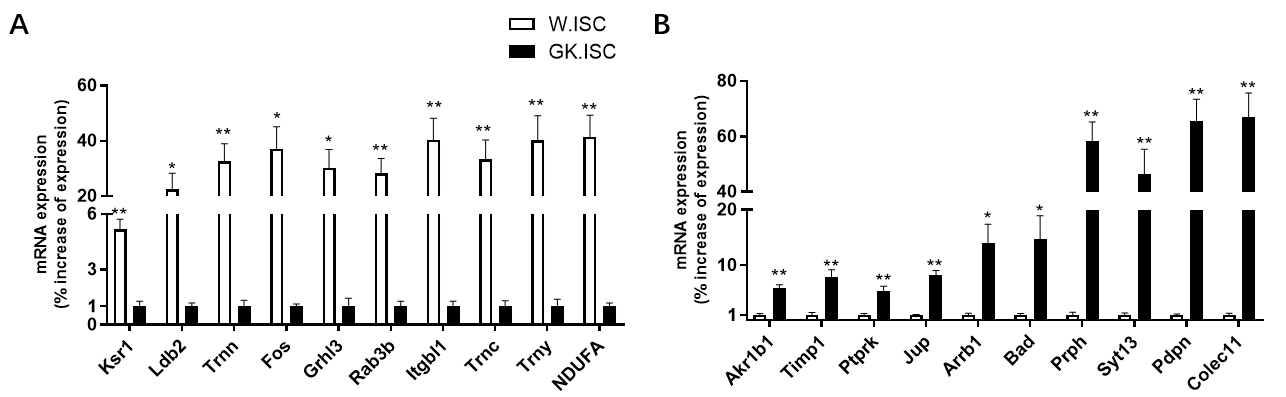

Supplement: Supplementary file 5 [file Image_2.jpeg]

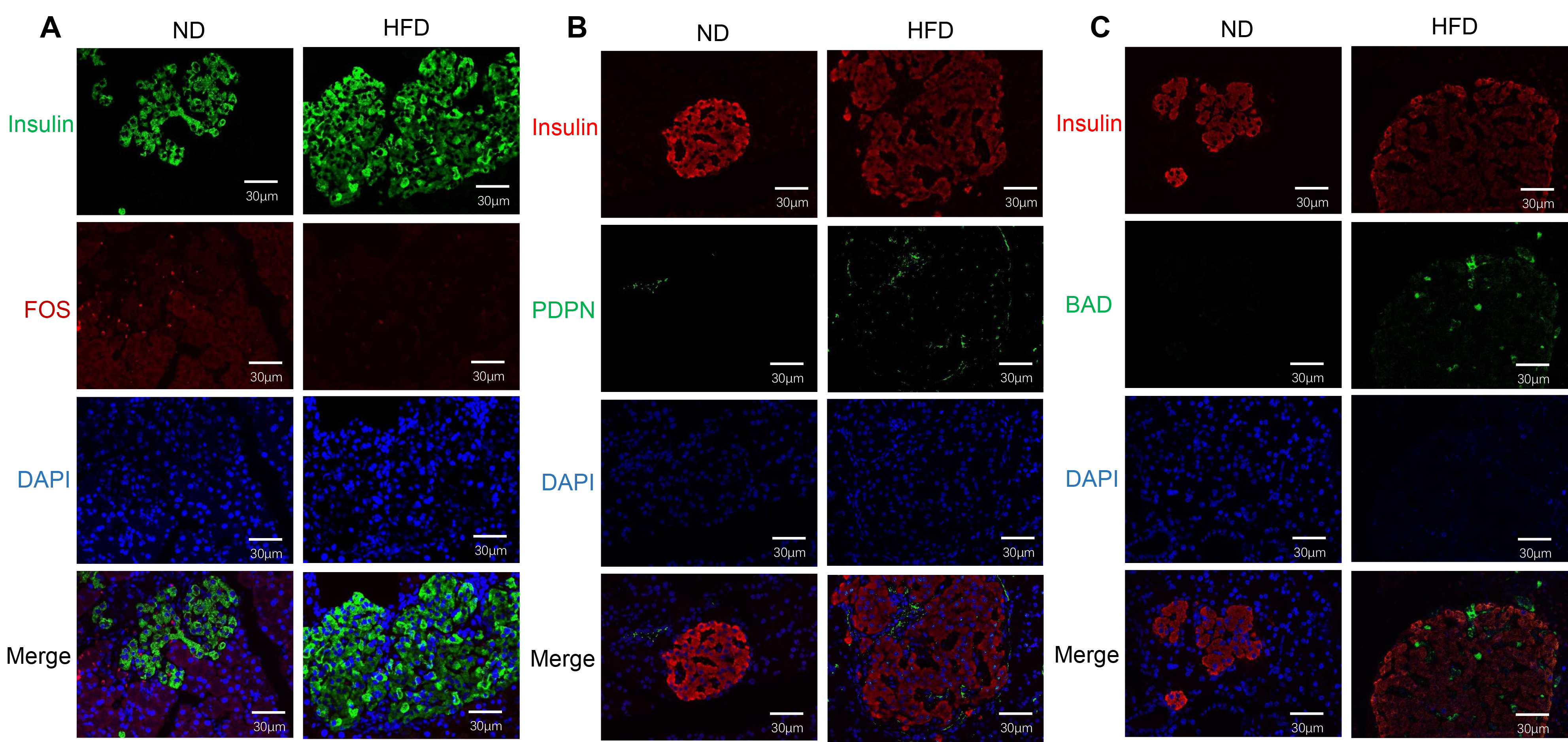

Supplement: Supplementary file 6 [file Image_3.jpg]
